# Complementary Color Tuning by HCl via Phosphorescence-to-Fluorescence Conversion on Insulated Metallopolymer Film and Its Light-Induced Acceleration

**DOI:** 10.3390/polym12010244

**Published:** 2020-01-20

**Authors:** Shunichi Kaneko, Hiroshi Masai, Takuya Yokoyama, Maning Liu, Yasuhiro Tachibana, Tetsuaki Fujihara, Yasushi Tsuji, Jun Terao

**Affiliations:** 1 Department of Basic Science, Graduate School of Arts and Sciences, The University of Tokyo, Tokyo 153-8902, Japan; kaneko-shun-ichi@g.ecc.u-tokyo.ac.jp (S.K.); cmasai.h@mail.ecc.u-tokyo.ac.jp (H.M.); 2 Department of Energy and Hydrocarbon Chemistry, Graduate School of Engineering, Kyoto University, Nishikyo-ku, Kyoto 615-8510, Japan; kyo.fruitful_tyu@me.com (T.Y.); tfuji@scl.kyoto-u.ac.jp (T.F.); ytsuji@scl.kyoto-u.ac.jp (Y.T.); 3 School of Engineering, RMIT University, Bundoora, Victoria 3083, Australia; eastoxford@msn.com (M.L.); yasuhiro.tachibana@rmit.edu.au (Y.T.)

**Keywords:** rotaxane, conjugated polymer, cyclodextrin, platinum acetylide, phosphorescence, fluorescence, white emission

## Abstract

An insulated metallopolymer that undergoes phosphorescence-to-fluorescence conversion between complementary colors by an acid-stimulus is proposed as a color-tunable material. A Pt-based phosphorescent metallopolymer, where the conjugated polymeric backbone is insulated by a cyclodextrin, is depolymerized by HCl via acidic cleavage of Pt-acetylide bonds to form a fluorescent monomer. The insulation enables phosphorescence-to-fluorescence conversion to take place in the solid film. Rapid color change was achieved by accelerating the reaction between the metallopolymer and HCl by UV irradiation. These approaches are expected to provide new guidelines for the development of next-generation color-tunable materials and printable sensors based on precise molecular engineering.

## 1. Introduction

**Figure 1:**
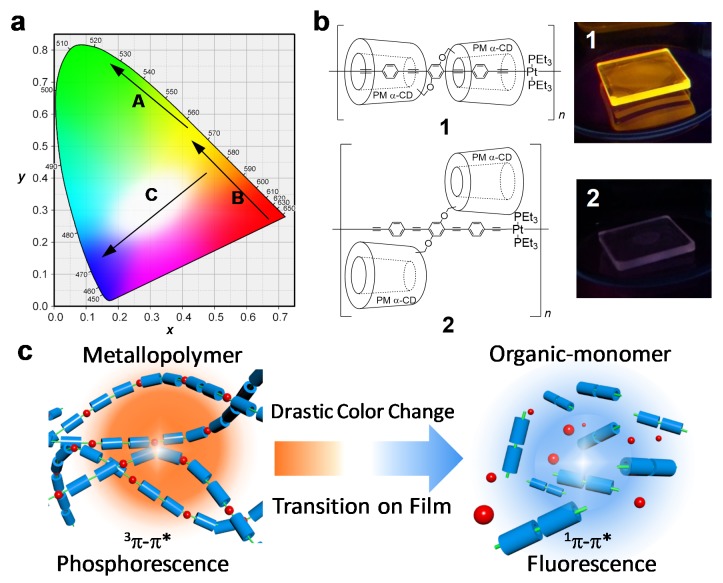
(**a**) Chromaticity diagram with arrows showing the yellow-to-green (A), red-to-yellow (B), and orange-to-white-to-blue (C) shifts. (**b**) Chemical structures and the photographic images (excitation at 365 nm) of emission under deoxygenated conditions for polymer **1** and polymer **2** films on SiO_2_ substrates. (**c**) Conceptual illustrations of phosphorescence-to-fluorescence conversion between complementary colors, triggered by metallopolymer to monomer conversion (This study).

Color-tunable luminescent materials by external stimuli have attracted considerable attention because of their potential for applications to chemical sensors [1,2,3] and security technologies [4,5]. Especially, polymeric materials have been considered practical because they are readily fabricated into films with high thermal stability and non-volatility [6,7]. Considering these applications, a drastic color change would be favorable for good visibility. Compared to the gradual color change between the adjacent color phases such as yellow-to-green and red-to-yellow (Figure 1a, arrows A and B, respectively), the color change between the complementary colors (orange-to-blue, Figure 1a, arrow C) signifies a dramatic change in the emission wavelength, via white midway through color shift [8]. Therefore, this system should improve the visibility of sensors or other security signals [9] and provide white emission materials.

Although the combination of two complementary colors to realize white emission has been achieved by mixing two molecular luminescences [10], controlling the emission colors from a single molecule is considered to be a simple and practical method [11,12,13,14,15,16,17,18]. In this work, a phosphorescence-to-fluorescence conversion (PFC) was selected as the color-changing mechanism because it provides a sufficient shift in the emission wavelength due to the large energy difference between the ^3^π–π* and ^1^π–π* transitions [16,17,18]. PFC can also be simply controlled by the detachment of heavy atoms on the luminescent π-conjugated system [19,20]. Nevertheless, PFC-based color-tunable materials are difficult to apply in practical polymer films because their emission can be quenched in the solid state due to the molecular interaction [21]. This problem should be addressed in order to develop design guidance for color-tunable materials based on PFC.

In our previous studies [22,23,24,25,26,27,28], we overcame the drawback of phosphorescent polymeric materials using a supramolecular approach. The platinum acetylide polymer backbones were completely covered with permethylated α-cyclodextrins (PM α-CDs), inhibiting molecular interactions between the adjacent conjugated chains (Figure 1b) [24]. Therefore, the phosphorescence of the polymer film was efficiently enhanced by insulating the platinum acetylide polymer (Figure 1b, the upper photo). We have also applied this insulation strategy to other polymer materials such as a luminescent sensor for typical metal ions [26], a HCl-responsive polymer utilizing acid-induced isomerization [27], and phosphorescent hydrogels [28]. In the present work, we developed a color-tunable material exhibiting white emission via the depolymerization of insulated Pt acetylide polymer that undergoes a PFC. For the external stimulation of depolymerization, we selected hydrogen chloride (HCl) as an acidic stimulus because Shaw and co-workers reported the chemical reaction between Pt-acetylide and HCl involving Pt−C bond cleavage [29]. The orange phosphorescent emission (^3^π–π*) of the insulated polymer due to the heavy atom effect [30] changes to the blue fluorescent emission (^1^π–π*) when HCl cleaves the Pt−C bonds (Figure 1c). Accordingly, we would provide a color-tunable polymer film based on PFC via white emission.

## 2. Materials and Methods 

### 2.1. Materials

Unless otherwise stated, commercially available chemicals were used as received. Reaction solvents were degassed through argon or nitrogen bubbling, before use. Polymer **1** [31], polymer **2** [31], and monomer **3** [32] were prepared according to our previous reports.

### 2.2. Synthesis of ***4***


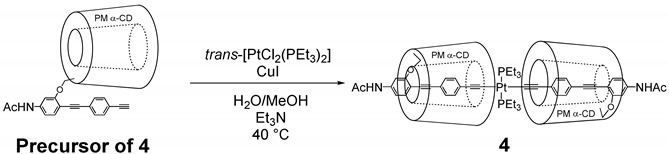


**Precursor of 4** [33] (100 mg, 68.1 μmol) and *trans*-PtCl_2_(PEt_3_)_2_ (16.4 mg, 32.7 μmol) were added into degassed MeOH/H_2_O (1/1, 100 mL), and the mixture was stirred at 40 °C for 30 min. Under a nitrogen atmosphere, degassed Et_3_N (3 mL) and CuI (0.7 mg, 3 μmol) were added into the solution, and then the reaction mixture was stirred at 40 °C for 18 h. The mixture was quenched with NH_4_Cl aq. and diluted with CHCl_3_. The organic layer was separated and dried over MgSO_4_, and then filtered. The solvent was removed by evaporation, the mixture was dried in vacuo and the residue was purified by GPC with CHCl_3_ as the eluent to yield **4** as a pale yellow solid (72 mg, 63 %). *ESI HR-MS*: (*m/z*) 1705.7249 ([**4** + Na_2_]^2+^, C_154_H_238_N_2_Na_2_O_62_P_2_Pt^2+^, calcd. 1705.7237). *^1^H NMR (500 MHz, CDCl_3_, r.t.)*: *δ*_H_ = 7.90 (d, *J* = 8.19 Hz, 4H, ArH), 7.42–7.30 (m, 10H, ArH and NH), 7.19 (s, 2H, ArH), 5.20–4.70 (m, 12H, CD-H), 4.40–2.80 (m, 174H, CD-H, OCH_3_), 2.20 (s, 6H, CH_3_), 2.10–1.95 (m, 12H, PCH_2_), 1.20–1.05 (m, 18H, PCH_2_CH_3_) (Supplementary Figure S15). *^13^C NMR* *(126 MHz, CDCl_3_, r.t.)*: *δ*_C_ = 168.24, 162.26, 139.29, 133.42, 132.16, 132.06, 130.49, 130.40, 129.44, 129.43, 118.55, 114.20, 112.84, 112.46, 100.76, 100.43, 100.08, 100.04, 99.90, 98.07, 94.27, 86.81, 83.84, 82.74, 82.63, 82.50, 82.39, 82.34, 82.32, 82.28, 82.14 (several peaks overlapped), 82.07 (several peaks overlapped), 81.71, 81.55, 81.22 (several peaks overlapped), 81.16, 81.03 (several peaks overlapped), 76.22, 72.08, 71.83, 71.70, 71.64 (several peaks overlapped), 71.26 (several peaks overlapped), 71.13 (several peaks overlapped), 71.11, 70.74, 70.17, 61.93, 61.81 (several peaks overlapped), 61.67, 61.59 (several peaks overlapped), 59.09 (several peaks overlapped), 58.96, 58.72, 58.69, 58.22, 57.91, 57.74, 57.66, 57.56, 57.47, 24.78, 16.02 (t, *J* = 17.5 Hz), 7.90 (Supplementary Figure S16). *^31^P NMR (202 MHz, CDCl_3_, r.t.)*: *δ*_P_ = 10.83 (s and d, ^1^*J*_P-Pt_ = 2369 Hz) (Supplementary Figure S17).

### 2.3. General Procedure of HCl Depolymerizing Experiment in the Solution State

Under air, insulated (**1**) polymer (0.25 mg) was dissolved in MeOH/H_2_O (2/1) (total volume: 1.0 mL) with hydrochloric acid, and then the mixture was stirred at room temperature. After the reaction, the reaction was quenched with NaHCO_3_ and then the solvent was removed by evaporation for the following analyses.

### 2.4. General Procedure of Depolymerizing Experiment in the Solid State

Insulated (**1**) or uninsulated (**2**) polymer film was fabricated by drop-casting or spin-casting of the polymer solution in CHCl_3_ (5 mg/mL) on SiO_2_ substrates (1 cm × 1 cm or 0.6 cm × 0.6 cm). The flask with the substrates was filled with HCl gas and other gases and was kept at room temperature. After the reaction, the reaction was quenched in vacuo to remove the reaction gases for the following analyses.

## 3. Results and Discussion

### 3.1. Depolymerizing Reaction of Pt-Acetylide Polymer ***1*** and ***2***

**Figure 2:**
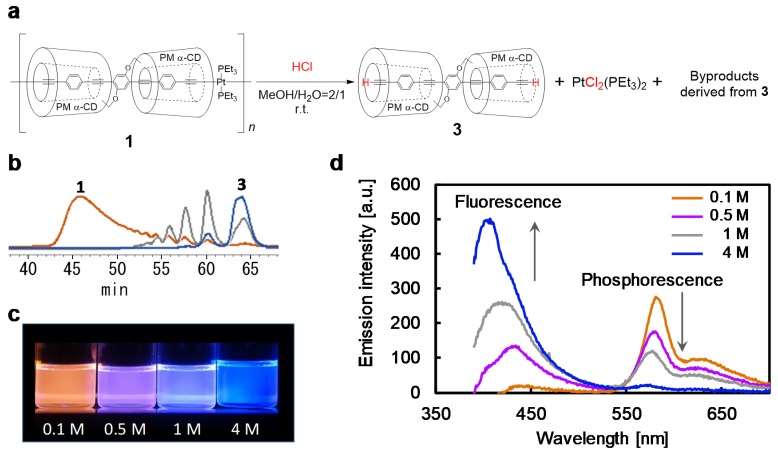
Depolymerizing experiments in the solution state: (**a**) Reaction of polymer **1** with HCl. (**b**) SEC analyses (detected at UV 380 nm) of the reaction products of polymer **1** before reaction (orange) and after reaction with 1 M HCl (gray) and 4 M HCl (blue) for 18 h. (**c**) Photographic images and (**d**) emission spectra of the reaction mixture of **1** by varying the concentration of HCl in (**c**), under deoxygenated conditions (excitation at 365 nm).

The metallopolymer **1** displayed orange phosphorescence (λ_max_ = 585 nm) in deoxygenated solution. Here, a solution of 4 M HCl in MeOH/H_2_O (2:1 *v*/*v*) afforded a cleavage on the Pt–acetylide bonds on the polymer **1** (Figure 2a). The depolymerization of **1** was confirmed by size exclusion chromatography (SEC) (Figure 2b). ^31^P NMR analysis of the depolymerizing mixture demonstrated the formation of monochlorinated and dichlorinated platinum complexes (Supplementary Figure S1) [34,35]. This result indicated that the Pt–acetylide bonds of the polymer **1** were cleaved in response to HCl. Furthermore, the photoluminescent behavior changed owing to the dissociation of the Pt atoms from the conjugated backbone. Under 365-nm excitation, the solution of the depolymerized mixture exhibited blue fluorescence (λ_max_ = 407 nm). The fluorescent peak-top wavelength of the depolymerized mixture corresponded to that of monomer bearing terminal alkynes (**3**) (Supplementary Figure S2). However, the spectrum of the mixture also accompanied a slight shoulder in the long wavelength region, which was possibly derived from the overreaction of the alkyne monomer to alkene derivatives.

### 3.2. Color-Tunability in the Solution State

In the presence of 1 M HCl, the solution displayed white emission. The color changes (orange-white-blue) reinforced the color distinctions even at concentrations that were only slightly different (0.1 M, 0.5 M, and 4 M) (Figure 2c,d). The quantum yield (QY) values of the phosphorescence and fluorescence after reaction with 0.1 M HCl were 15.6% and 0.8%, respectively. On the other hand, after reaction with 4 M HCl, the QY of phosphorescence decreased to 2.7% and that of fluorescence increased to 8.5% (Supplementary Table S1). The time course of the reaction with the 4 M HCl solution showed a similar spectrum change as that observed in Figure 2d (Supplementary Figure S4). Thus, the orange-white-blue color change occurred during the depolymerization of **1** with HCl. Accordingly, PFC after depolymerization indicated that polymer **1** had great potential as a color-tunable material of HCl based on phosphorescence.

### 3.3. Depolymerizing Reaction in the Solid State

**Figure 3:**
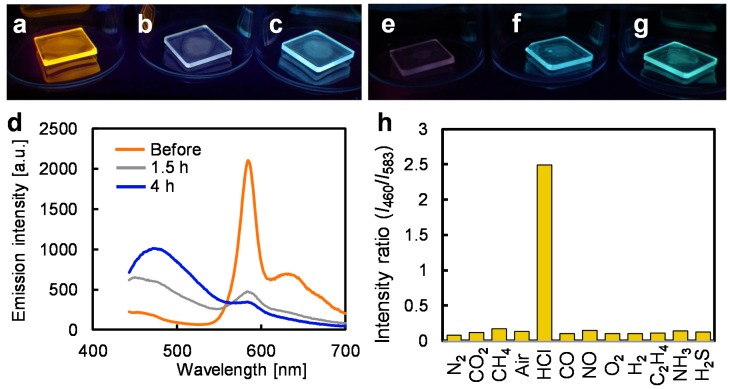
Depolymerizing experiments in the solid state: Emission photographs under deoxygenated conditions (excitation at 365 nm) of polymer **1** films on SiO_2_ substrates (**a**) before reaction, and after reaction with HCl gas for (**b**) 1.5 h, and (**c**) 4 h. (**d**) Emission spectra of polymer **1** film in (**a**–**c**). Emission photographs of polymer **2** films (**e**) before reaction, and after reaction with HCl gas for (**f**) 10 min, and (**g**) 30 min, under the same conditions as (**a**–**c**). (**h**) Emission intensity ratio between 460 nm and 583 nm (excitation at 365 nm) of polymer **1** films on SiO_2_ substrates in the presence of various gases after 3 h under deoxygenated conditions.

Polymer films of **1** and **2** were fabricated by spin-casting a solution of the polymer in CHCl_3_ onto SiO_2_ substrates. Under irradiation at 365 nm, the polymer film of **1** showed orange phosphorescence identical to that in solution (Figure 3a,d, orange line). After exposure to 1 atm HCl gas for 1.5 h, the emission exhibited a remarkable color change to white on the film (Figure 3b,d gray line). Accordingly, the PFC between complementary colors was triggered by HCl even at the solid-gas interface. Further reaction with HCl for 4 h afforded blue fluorescence (Figure 3c,d blue line). The emission intensity of the polymer **1** did not change after UV irradiation for several hours, indicating its stability (Supplementary Figure S6). On the other hand, the uninsulated counterpart **2** interacted with adjacent molecules in the solid state, such that phosphorescence was almost quenched, in contrast to **1** (Figure 3e). Therefore, during the intermediate stage of depolymerization, the uninsulated polymer film of **2** showed gradual color change from red to green (Figure 3e–g and Figure S8) without white emission. These results indicate that insulation is essential for the application of color-tunable polymer films.

### 3.4. Chemospecific Reactivity for Pt–Acetylide Bonds with HCl Gas

The reaction selectivity of the polymer film of **1** was evaluated by measuring the optical response in the presence of various gases after 3 h. The vertical axis in Figure 3h shows the ratio of emission intensity of the fluorescence at 460 nm and phosphorescence at 583 nm after exposure to each gas shown on the horizontal axis. Apart from the HCl gas, no emission changes were observed in the presence of oxidative (NO and O_2_) and reductive (CO, H_2_ and C_2_H_4_) gases, in addition to inert gases such as N_2_, CO_2_, CH_4_, and air. Moreover, non-reactivity to a basic gas (NH_3_) and even to a weak acidic gas (H_2_S) confirmed the chemospecific reaction of polymer **1** for depolymerization with HCl. The selectivity would also indicate the high potential of insulated polymer **1** for applications to optochemical HCl gas sensors in the solid state [36]. Notably, the polymer **1** could be used even under ambient conditions because the phosphorescence is not affected by oxygen due to the insulation [24]. Finally, PFC was confirmed within less than 5 s after the spraying of HCl gas (5 mL) onto the polymer film of **1** under ambient conditions, realizing real-time HCl gas detection (Supplementary Figure S11 and Movie S1).

### 3.5. Light-Induced Acceleration for the Depolymerization

**Figure 4:**
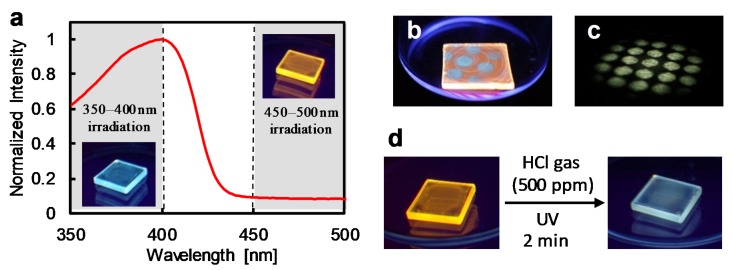
Experimental results of light-induced acceleration of polymer **1** film on SiO_2_ substrate: (**a**) Photographic images (upon 365-nm excitation under deoxygenated conditions): Resultant substrate after 5% *v*/*v* HCl exposure for 5 min with UV irradiation of each wavelength range and an absorption spectrum of **1** in dilute CHCl_3_. (**b**) Photographic images (upon 365-nm excitation under deoxygenated conditions) of the resultant film with spot irradiations (350–400 nm) and 10% *v*/*v* HCl gas. (**c**) Photographic image of irradiation pattern in (**b**) with white light on black paper. (**d**) Photographic images (upon 365-nm excitation under deoxygenated conditions) before and after reaction with 500-ppm HCl gas for 2 min using light-induced acceleration.

The low reaction rate at low concentration is an intrinsic disadvantage of the color-tunable materials based on the chemical reaction. In contrast, highly reactive materials are unstable under ambient conditions and do not permit long-term storage, whereas stable stimuli-responsive materials intrinsically afford slow responses. Thus, a tradeoff relationship exists between reactivity and stability. We successfully overcame this problem by the photo-excitation of the π-conjugated backbone in the presence of HCl gas. The UV irradiation in the 350–400 nm range accelerated the depolymerization reaction in the presence of 5% *v*/*v* HCl gas (left-hand side image in Figure 4a and Figure S12d), and the resultant film displayed blue fluorescence. On the other hand, irradiation in the 450–500 nm range did not affect the reaction rate, because the emission color after UV irradiation remained unchanged (right-hand side image in Figure 4a and Supplementary Figure S12c). This result indicated that the wavelength of irradiation for the acceleration needed to correspond with that of the absorption band of the polymer **1** (Figure 4a). In addition, without irradiation, acceleration of the reaction at 100 °C was negligible, which demonstrated that acceleration could not be attributed to local heating caused by irradiation (Supplementary Figure S12b). UV irradiation of specific areas selectively converted the polymer **1** in these areas into monomers, indicating that the accelerated reaction required the concerted action of the HCl gas and UV irradiation (Figure 4b,c). Finally, the film was exposed to 500 ppm of HCl gas for 2 min under UV irradiation. Compared to the ambient conditions that provide white emission without irradiation (Figure 3b), the gas concentration and reaction time were approximately 2000 times lower and 50 times shorter, respectively, due to light-induced acceleration. The light-induced acceleration was also observed in the dilute solution system of insulated polymer **4** in MeOH/H_2_O solution with 5000 ppm HCl and 365 nm UV irradiation. See Figure S14.

**Figure 5:**
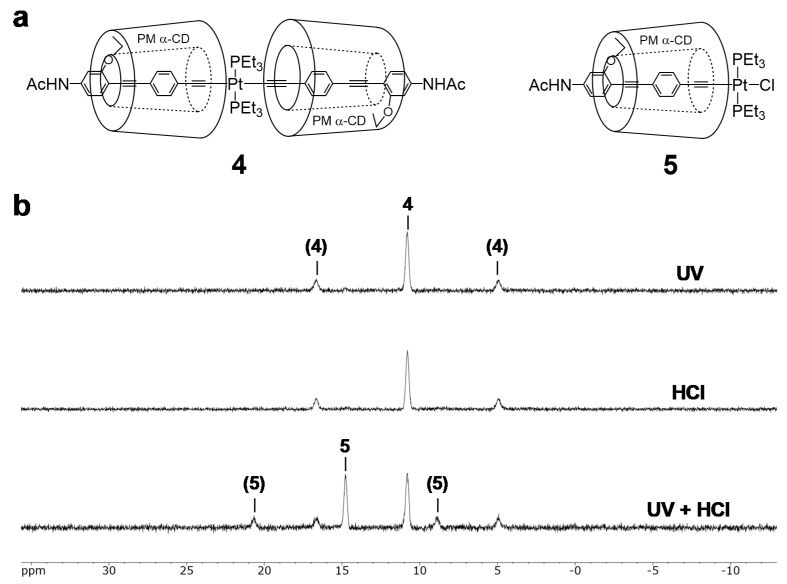
Light-induced acceleration of Pt-acetylide complex **4**: (**a**) Chemical structure of **4** and monochlorinated Pt complex **5**. (**b**) ^31^P NMR (202 MHz, CDCl_3_) analyses of **4** after reaction for 10 min under UV, HCl, and UV + HCl. Coupling peaks with platinum atom are in parentheses.

In order to further investigate the light-induced effects, an Pt-acetylide complex **4** was prepared as a partial structure of polymer **1** (Figure 5a). The reaction was confirmed in the DMF solution and its progress was characterized by ^31^P NMR analyses. As a result, 47% of **4** underwent cleavage of the Pt-acetylide bond to form monochlorinated Pt complex **5** under UV radiation (365 nm) in the presence of hydrochloric acid (1 M) after 20 min (Figure 5b) [35]. On the other hand, **4** was intact under the single stimulus, either by UV irradiation or by the addition of HCl. The monomer experiment clearly demonstrated that the UV irradiation accelerated the reaction between the Pt-acetylide and HCl. This approach provides a new design strategy to improve the tradeoff relationship in the stimuli-responsive materials in the point of stability and reactivity.

## 4. Conclusions

In summary, the insulated Pt-acetylide polymer emerged as a new material for application in color-tunable materials. The acidic stimulus (HCl) cleaved the Pt-acetylide bonds, which afforded the luminescent conversion between complementary colors. The large wavelength-shift between phosphorescence and fluorescence enabled color-tunability involving white emission. The material possessed unique properties such as insulation effects for utilization in the polymer films and light-induced acceleration for improving reactivity. The molecular design demonstrated great potential for use as next-generation printable and white-emission materials and chemical sensors due to its facile preparation.

## Supplementary Materials

The following are available online at https://www.mdpi.com/2073-4360/12/1/244/s1, Figure S1. ^31^P NMR spectrum of the depolymerizing mixture of 1, Figure S2. Emission spectra of depolymerized mixtures of 1 and of 3, Figure S3. SEC analyses of 1 with various HCl concentration, Table S1. The QY after the depolymerization of 1, Figure S4. (a) Time-course of emission spectra of the reaction mixture of 1 with HCl. (b) Photographic images of the emission behavior of the reaction mixture of 1 with HCl, Figure S5. Emission spectra of 1 before reactions with HCl gas, Figure S6. Emission spectra of 1 in CHCl_3_ before irradiation and after UV irradiation, Figure S7. SEC analyses of 1 after reactions with HCl gas and of 2 after reactions with HCl gas, Figure S8. Emission spectra of polymer films of 2 before and after reaction with HCl gas, Figure S9. Emission spectra of 1 after reactions with various gases, Figure S10. SEC analyses of 1 after reactions with various gases, Figure S11. (a) Photographic images of polymer films of 1 film before and after the reaction with HCl gas spray. (b) Illustration of the experiment, Figure S12. SEC analyses of 1 under HCl gas with various reaction conditions, Figure S13. (a) Emission spectra and (b) photographic images of the reaction mixture of 1 before and after reaction with 5000 ppm HCl under UV irradiation, Figure S14. Emission spectra of the reaction mixture of 1 before and after reaction with 500 ppm HCl under UV irradiation, Figure S15. ^1^H NMR spectrum of 4, Figure S16. ^13^C NMR spectrum of 4, Figure S17. ^31^P NMR spectrum of 4, Movie S1. Real-time HCl gas detection.

## References

[1] Lim S.H., Feng L., Kemling J.W., Musto C.J., Suslick K.S. (2009). An optoelectronic nose for the detection of toxic gases. Nat. Chem..

[2] Wenger O.S. (2013). Vapochromism in Organometallic and Coordination Complexes: Chemical Sensors for Volatile Organic Compounds. Chem. Rev..

[3] Ma D.-L., Ma V.P.-Y., Chan D.S.-H., Leung K.-H., He H.-Z., Leung C.-H. (2012). Recent advances in luminescent heavy metal complexes for sensing. Coord. Chem. Rev..

[4] Yoon B., Lee J., Park I.S., Jeon S., Lee J., Kim J.-M. (2013). Recent functional material based approaches to prevent and detect counterfeiting. J. Mater. Chem. C.

[5] Hou X., Ke C., Bruns C.J., McGonigal P.R., Pettman R.B., Stoddart J.F. (2015). Tunable solid-state fluorescent materials for supramolecular encryption. Nat. Commun..

[6] Guan W., Zhou W., Lu J., Lu C. (2015). Luminescent films for chemo- and biosensing. Chem. Soc. Rev..

[7] Sun X., Chen T., Huang S., Li L., Peng H. (2010). Chromatic polydiacetylene with novel sensitivity. Chem. Soc. Rev..

[8] Pridmore R.W. (2011). Complementary colors theory of color vision: Physiology, color mixture, color constancy and color perception. Color Res. Appl..

[9] Heo J.H., Cho H.H., Leea J.W., Lee J.H. (2014). Achromatic-chromatic colorimetric sensors for on-off type detection of analytes. Analyst.

[10] Kido J., Kimura M., Nagai K. (1995). Multilayer white light-emitting organic electroluminescent device. Science.

[11] Fleetham T., Li G., Li J. (2017). Phosphorescent Pt(II) and Pd(II) Complexes for Efficient, High-Color-Quality, and Stable OLEDs. Adv. Mater..

[12] Park S., Kwon J.E., Kim S.H., Seo J., Chung K., Park S.-Y., Jang D.-J., Medina B.M., Gierschner J., Park S.Y. (2009). White-Light-Emiting Molecule: Frustrated Energy Transfer between Constituent Emitting Centors. J. Am. Chem. Soc..

[13] Fleetham T., Ecton J., Wang Z., Bakken N., Li J. (2013). Single-doped white organic light-emitting device with an external quantum efficiency over 20%. Adv. Mater..

[14] Chen P., Li Q., Grindy S., Holten-Andersen N. (2015). White-Light-Emitting Lanthanide Metallogels with Tunable Luminescence and Reversible Stimuli-Responsive Properties. J. Am. Chem. Soc..

[15] Yamaguchi K., Murai T., Guo J.D., Sasamori T., Tokitoh N. (2016). Acid-Responsive Absorption and Emission of 5-*N*-Arylaminothiazoles: Emission of White Light from a Single Fluorescent Dye and a Lewis Acid. ChemistryOpen.

[16] Radotić K., Melø T.B., Leblanc R.M., Yousef Y.A., Naqvi K.R. (2016). Fluorescence and phosphorescence of tryptophan in peptides of different length and sequence. J. Photochem. Photobiol. B Biol..

[17] Xu R., Wang Y., Duan X., Lu K., Micheroni D., Hu A., Lin W. (2016). Nanoscale Metal–Organic Frameworks for Ratiometric Oxygen Sensing in Live Cells. J. Am. Chem. Soc..

[18] Zang L., Zhao H., Hua J., Qin F., Zheng Y., Zhang Z., Cao W. (2016). Ratiometric dissolved oxygen sensitive indicator based on lutetium labeled hematoporphyrin monomethyl ether with balanced phosphorescence and fluorescence dual emission. Sens. Actuators B Chem..

[19] Wu H., Hang C., Li X., Yin L., Zhu M., Zhang J., Zhou Y., Ågren H., Zhang Q., Zhu L. (2017). Molecular stacking dependent phosphorescence–fluorescence dual emission in a single luminophore for self-recoverable mechanoconversion of multicolor luminescence. Chem. Commun..

[20] Mao Z., Yang Z., Mu Y., Zhang Y., Wang Y.F., Chi Z., Lo C.C., Liu S., Lien A., Xu J. (2015). Linearly Tunable Emission Colors Obtained from a Fluorescent-Phosphorescent Dual-Emission Compound by Mechanical Stimuli. Angew. Chem. Int. Ed. Engl..

[21] Gong S., Yang C., Qin J. (2012). Efficient phosphorescent polymer light-emitting diodes by suppressing triplet energy back transfer. Chem. Soc. Rev..

[22] Terao J., Wadahama A., Matono A., Tada T., Watanabe S., Seki S., Fujihara T., Tsuji Y. (2013). Design principle for increasing charge mobility of π-conjugated polymers using regularly localized molecular orbitals. Nat. Commun..

[23] Masai H., Terao J., Seki S., Nakashima S., Kiguchi M., Okoshi K., Fujihara T., Tsuji Y. (2014). Synthesis of One-Dimensional Metal-Containing Insulated Molecular Wire with Versatile Properties Directed toward Molecular Electronics Materials. J. Am. Chem. Soc..

[24] Masai H., Terao J., Makuta S., Tachibana Y., Fujihara T., Tsuji Y. (2014). Enhancement of Phosphorescence and Unimolecular Behavior in the Solid State by Perfect Insulation of Platinum—Acetylide Polymers. J. Am. Chem. Soc..

[25] Masai H., Terao J. (2017). Stimuli-responsive functionalized insulated conjugated polymers. Polym. J..

[26] Hosomi T., Masai H., Fujihara T., Tsuji Y., Terao J. (2016). A Typical Metal-Ion-Responsive Color-Tunable Emitting Insulated π-Conjugated Polymer Film. Angew. Chem. Int. Ed..

[27] Miyagishi H.V., Tamaki T., Masai H., Terao J. (2019). Synthesis and Acid-Responsiveness of an Insulatedπ-Conjugated Polymer Containing Spiropyrans in Its Backbone. Molecules.

[28] Russell G.M., Inamori D., Masai H., Tamaki T., Terao J. (2019). Luminescent and mechanical enhancement of phosphorescent hydrogel through cyclic insulation of platinum-acetylide crosslinker. Polym. Chem..

[29] Perera S.D., Shaw B.L. (1991). Complexes including acetylides formed from 3-diphenylphosphinocamphor and platinum or palladium. J. Organomet. Chem..

[30] Liu Y., Jiang S., Glusac K., Powell D.H., Anderson D.F., Schanze K.S. (2002). Photophysics of Monodisperse Platinum-Acetylide Oligomers: Delocalizaton in the Singlet and Triplet Excited States. J. Am. Chem. Soc..

[31] Terao J., Masai H., Fujihara T., Tsuji Y. (2012). Synthesis of Insulated Pt—Alkynyl Complex Polymer. Chem. Lett..

[32] Terao J., Tsuda S., Tanaka Y., Okoshi K., Fujihara T., Tsuji Y., Kambe N. (2009). Synthesis of Organic-Soluble Conjugated Polyrotaxanes by Polymerization of Linked Rotaxanes. J. Am. Chem. Soc..

[33] Masai H., Terao J., Fujihara T., Tsuji Y. (2016). Rational design for rotaxane synthesis through intramolecular slippage: Control of activation energy by rigid axle length. Chem. A Eur. J..

[34] Rahn J.A., Nelson J.H., Baltusis L. (1999). Solid-state structures of platinum phosphine, (R_3_P)_2_PtX_2_ complexes as determined by a combination of ^13^C{^1^H} and ^31^P{^1^H} NMR spectroscopy. Inorg. Chem..

[35] Sebald A., Stader C., Wrackmeyer B., Bensch W. (1986). Alkynyl/chloride exchange between trans-platinum(II) and -palladium(II) chlorides and alkynylstannanes. Crystalstructure of trans-[bis(1-propynyl)-bis(triethylphosphine)platinum(II)]. J. Organomet. Chem..

[36] Zhou X., Lee S., Xu Z., Yoon J. (2015). Recent Progress on the Development of Chemosensors for Gases. Chem. Rev..

