# Supplementary material for: Complementary Color Tuning by HCl via Phosphorescence-to-Fluorescence Conversion on Insulated Metallopolymer Film and Its Light-Induced Acceleration
Source: Polymers (Basel). 2020 Jan 20;12(1):244. doi: 10.3390/polym12010244 (PMC7023531; doi:10.3390/polym12010244)
Supplement: Supplementary file 1 [file polymers-12-00244-s001.zip › polymers-685178.SI.proof done/Proofreading Supplementary Materials3.pdf]

Supplementary Materials

Article

Complementary Color Tuning by HCl via Phosphorescence-to-Fluorescence Conversion on Insulated Metallopolymer Film and Its Light-Induced Acceleration

Shunichi Kaneko [1], Hiroshi Masai [1], Takuya Yokoyama [2], Maning Liu [3], Yasuhiro Tachibana [3], Tetsuaki Fujihara [2], Yasushi Tsuji [2] and Jun Terao [1,*]

[1] Department of Basic Science, Graduate School of Arts and Sciences, The University of Tokyo, Tokyo 153-8902, Japan; kaneko-shun-ichi@g.ecc.u-tokyo.ac.jp (S.K.); cmasai.h@mail.ecc.u-tokyo.ac.jp (H.M.)

[2] Department of Energy and Hydrocarbon Chemistry, Graduate School of Engineering, Kyoto University, Nishikyo-ku, Kyoto 615-8510, Japan; kyo.fruitful_tyu@me.com (T.Y.); tfuji@scl.kyoto-u.ac.jp (T.F.); ytsuji@scl.kyoto-u.ac.jp (Y.T.)

[3] School of Engineering, RMIT University, Bundoora, Victoria 3083, Australia; eastoxford@msn.com (M.L.); yasuhiro.tachibana@rmit.edu.au (Y.T.)

* Correspondence: cterao@mail.ecc.u-tokyo.ac.jp

Table of Contents

1. General Remarks S3
2. Experimental Procedures and Supplimentary Results
2-1. Depolymerizing experiments in the solution S5
^{31}P NMR analysis of depolymerizing mixture
Emission spectra of depolymerizing mixture
Concentration dependence
The stability of polymer 1 under UV light
Time-course of the reaction
2-2. Depolymerizing experiments in the solid state S9
Reproducibility of preparation of polymer film
Time-course of depolymerization
Gas selectivity
HCl detection under ambient condition
2-3. Ligth-induced acceleration of HCl reaction S15
Control experiment
Site-selective irradiation
Experiments for low HCl concentration in the solution
Experiments for low HCl concentration in the solid state
^{31}P NMR analyses for complex 4
2-4. NMR spectra of 4 S18
3. References S21

1. General Remarks

NMR Spectroscopy: ^{1}H NMR (500 MHz), $^{13}C\{^{1}H\}$ NMR (126 MHz), and $^{31}P\{^{1}H\}$ NMR (202 MHz) were measured with a Bruker AVANCE-500 spectrometer. The ^{1}H NMR chemical shifts were reported relative to tetramethylsilane (TMS, 0.00 ppm) or residual protonated solvents (7.26 ppm) in $CDCl_3$. The ^{13}C NMR chemical shifts were reported relative to $^{13}CDCl_3$ (77.16 ppm). The ^{31}P NMR spectra were also recorded using 85% H_3PO_4 as an external standard.

High-Resolution Mass Spectroscopy (HR-MS): Electrospray ionization time-of-flight (ESI-TOF) mass spectra were obtained using a Waters Xev G2-S TOF mass spectrometer.

Preparative recycling gel permeation chromatography (GPC): Preparative recycling GPC was performed with a SHIMADZU LC-20AP System equipped with a Shodex K-4002.5L column, a SHIMADZU SPD-20A, and a SHIMADZU RID-10A using $CHCl_3$ as the eluent at a flow rate of 14 mL min^{-1}.

Analytical Size-exclusion Chromatography (SEC): Analytical SEC was performed with a GL-Science GL-7400 HPLC System equipped with Shodex KF-801, -802, -802.5, -803, -804 columns, a GL-7410 HPLC pump, a GL-7400 UV detector, and a GL-7454 RI detector using THF as the eluent at a flow rate of 0.6 mL min^{-1}.

Absorption/Emission Spectra: Solution samples were prepared in concentrations of 10^{-2} mg/mL. The solutions were degassed through argon or nitrogen bubbling for 30 min. The polymer films were fabricated by spin casting a solution of the polymer in $CHCl_3$ (5 mg/mL) onto SiO_2 substrates (1 cm × 1 cm or 0.6 cm × 0.6 cm). Absorption spectra were measured with a UV-Vis absorption spectrometer (SHIMADZU, UV-2450 or UV-2600). Photoluminescence spectra of solution samples were obtained using a HITACHI F-7000 fluorescence spectrophotometer model equipped with a 150 W xenon lamp and using a HAMAMATSU C11347 with a calibrated integrating sphere system. Photoluminescence spectra and quantum yield of samples were obtained using a Photon Technology International (PTI) fluorometer with a xenon arc lamp light source and with a spectral resolution of 4 nm at room temperature. The spectra were corrected for spectral response of a grating in an emission monochromator and a detector.

Gas Preparation: CH_4 (99.9% purity) and C_2H_4 (99.5% purity) gases were supplied by portable gas canisters which were purchased from GL Science. CO (99.995% purity), CO_2 (99.99%

purity), and H_2 (99.99% purity) gas cylinders were purchased from Sumitomo Seika Chemicals, Nippon Ekitan, and Kyoto Teisan, respectively. N_2 was prepared via vaporization of liquid N_2 purchased from Iwatani (99.995% purity) or ATOX (99.999% purity). O_2 was supplied by a gas cylinder which was purchased from Kyoto Teisan (99.5% purity) or by a portable gas canister which was purchased from GL Science (99.9% purity). Other gases were prepared via laboratory methods with following purification as described below.

HCl ($2NaCl + H_2SO_4 \rightarrow HCl + Na_2SO_4$) was passed through $CaCl_2$ powder to trap out H_2O contaminants.

NO ($3Cu + 8HNO_3$ (dil.) $\rightarrow 3\ Cu(NO_3)_2 + 2NO + 4H_2O$) was passed through water, $CaCl_2$ powder, and then KOH pellet to trap out NO_2, water and acidic gases, respectively.

NH_3 ($NaOH + NH_4Cl \rightarrow NH_3 + NaCl + H_2O$) was passed through KOH pellet to trap out water.

H_2S ($FeS + H_2SO_4$ (dil.) $\rightarrow H_2S + FeSO_4$) was passed through $CaCl_2$ powder to trap out H_2O contaminants.

Air was used after passing through $CaCl_2$ powder to trap out H_2O contaminants.

2. Experimental Proccedures and Supplementary Results

2-1. Depolymerizing experiments in the solution

^{31}P NMR analysis of depolymerizing mixture

A depolymerizing reaction of insulated polymer **1** (4.0 mg) with 3 M HCl in MeOH/H_2O (2/1, 2.0 mL) was conducted for 24 h. The reation was quenched with $NaHCO_3$ and then the solvent was removed by evaporation. The residue was dissolved in $CDCl_3$. The solution was filtered with a celite and the filtrate was analyzed by ^{31}P NMR at room temperature as shown in Figure S1.

The initial peak of insulated polymer at 11.86 ppm completely disappeared. The new peak at 12.19 ppm was identified as *trans*-[$PtCl_2(PEt_3)_2$] purchased form Aldrich. The peak at 9.05 ppm was identified as *cis*-[$PtCl_2(PEt_3)_2$] according to a reported literature [1]. The result supported that the insulated Pt–acetylide polymer **1** depolymerized into monomer **3** and $PtCl_2(PEt_3)_2$ in response to HCl. The peak at 14.60 ppm was estimated as mono-chlorinated platinum complex [2].

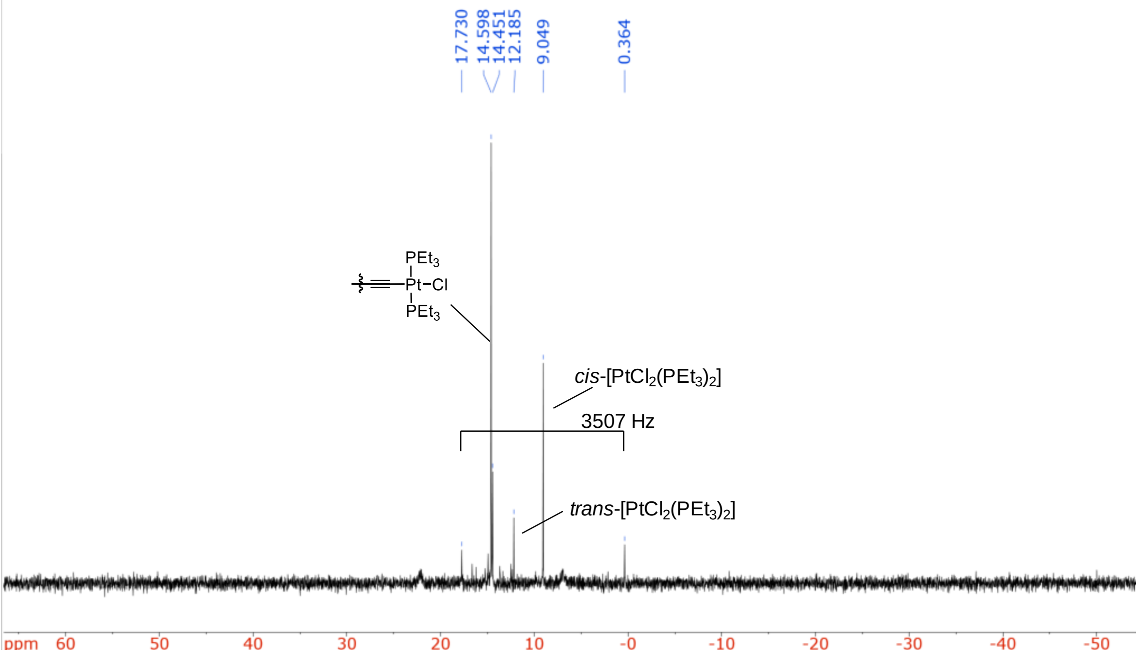

Figure S1. ^{31}P NMR spectrum (202 MHz, $CDCl_3$) of the depolymerizing mixture of metallopolymer **1**.

Emission spectra of depolymerizing mixture

Figure S2 displayed emission spectra of the fully depolymerized mixtures of **1** with 4 M HCl and of monomer **3** in MeOH/H_2O (2/1). The maximum emission wavelengths were identical between two samples as 407 nm, indicating the depolymerized mixtures probably included monomer **3**. However, the spectrum of the reaction mixture also accompanied a slight shoulder in the long wavelength region, which was possibly derived from the overreaction of the alkyne monomer to alkene derivatives.

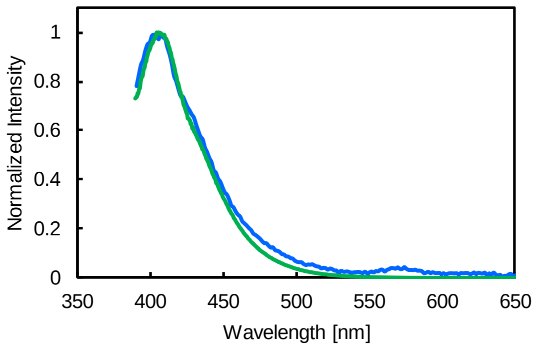

Figure S2. Emission spectra of depolymerized mixtures of **1** (blue) and of monomer **3** (green) in MeOH/H_2O (2/1).

Concentration dependence

Under air, insulated polymer (0.25 mg) was desolved in MeOH/H_2O (2/1) (total volume: 0.5 mL) with 12 M hydrochloric acid (4 μL, 21 μL, 42 μL, and 167 μL for 0.1 M, 0.5 M, 1 M, and 4 M, respectively), and then the mixture was stirred at room temperature for 18 h. Figure S3 indicated that the depolymerization was proceeded in accordance with high HCl concentration. The quantum yield (QY) of the solution after depolymerization was also measured (Table S1). The QY of phosphorescence decreased form 15.6 % to 2.7 % with the increase of HCl concentration, while that of fluorescence increased from 0.8 % to 8.5 %.

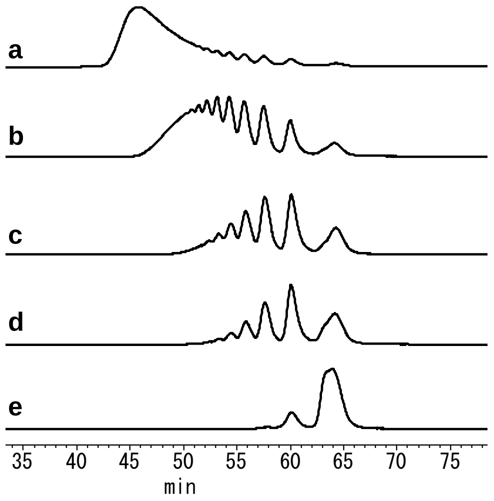

Figure S3. SEC analyses (detected: UV 380 nm) of the insulated polymer **1** with various HCl concentration; (a) before reaction , (b) 0.1 M, (c) 0.5 M, (d) 1 M, and (e) 4 M.

Table S1. The quantum yield (QY) after the depolymerization (λ_{ex} = 380 nm).

HCl concentration (M)	QY of phosphorescence (%)	QY of fluorescence (%)
0.1	15.6	0.8
0.5	12.8	2.6
1	9.4	5.0
4	2.7	8.5

Time-course of the reaction

Under air, insulated polymer **1** (0.25 mg) was desolved in MeOH/H_2O (2/1) (total volume: 0.5 mL) with 12 M hydrochloric acid (167 μL) to provide the desired concentrations (1 M) and then the mixture was stirred at room temperature. The emission behavior was monitored after 1.5 h, 9 h, and 18 h. Figure S4a showed the emission spectra of each solution, which demonstrated that the phosphorescence changed to fluorescence in the progress of the reaction. The emission color turned to white after 1.5 h and to blue after 18 h (Figure S4b).

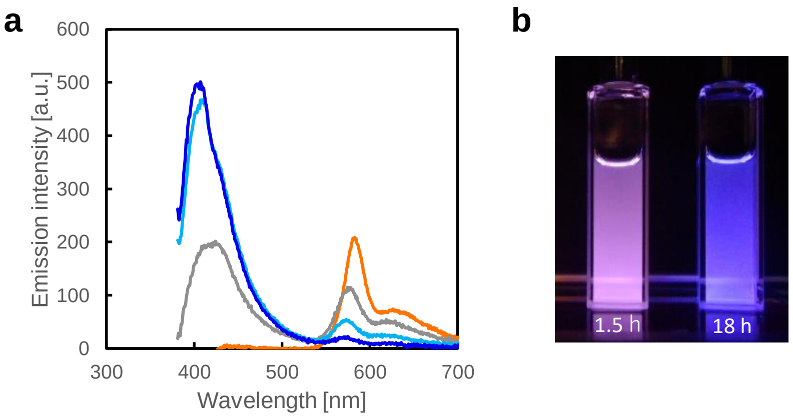

Figure S4. (a) Time-course of emission spectra of the reaction mixture in MeOH/H_2O (2/1) with insulated polymer **1** and HCl before raction (orange), after 1.5 h (gray), after 9 h (light blue), and after 18 h (blue). (b) Photographic images under 365 nm excitation of the emission behavior of the reaction mixture in deoxygenated MeOH/H_2O (2/1) (left) after 1.5 h and (right) after 18 h.

2-2. Depolymerizing experiments in the solid state

Reproducibility of preparation of polymer film

Spectroscopic analyses were conducted by utilizing spin-casted substrates for quantitative assessment. The reproducibility of the fabrication of the polymer films was confirmed by recording their emission spectra before reaction with HCl gas. Three insulated polymer (**1**) films were prepared by spin casting a solution of the polymer onto individual SiO_2 substrates according to the standard procedure. The emission spectra obtained under deoxygenated conditions all displayed maximum emission at the same wavelength and showed similar emission intensities (Figure S5). The results demonstrated that film fabrication via spin coating was highly reproducible as confirmed by the spectroscopic analyses.

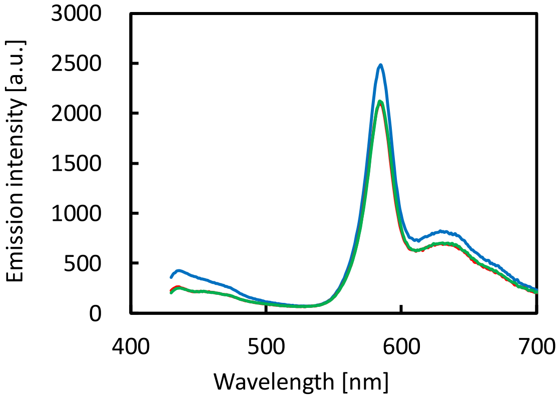

Figure S5. Three emission spectra (blue, green, and red) under deoxygenated condition of insulated polymer **1** on SiO_2 substrates before reactions with HCl gas.

The stability of polymer 1 under UV light

Polymer **1** (0.1 mg) on SiO_2 substarate (1 cm × 1 cm) was exposed to UV light for several hours under ambient condition in order to confirm the stability. UV irradiation was performed with SLUV-4 (365 nm, 4 W) at room temperature. After the irradiation, the polymer was dissolved in $CHCl_3$ and the solution was bubbled by N_2 gas for 20 min. The emission spectrum of the deoxygenized solution were measured. As a result, the emission intensity and the emission spectra after irradiation for 8 hours remained intact (Figure S6). This result indicates that the polymer has enough stability as a potential application.

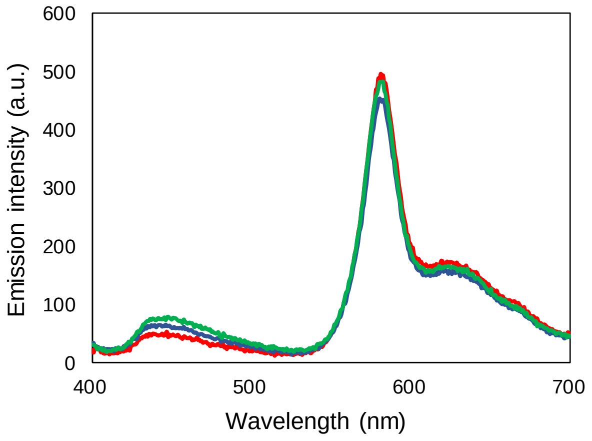

Figure S6. Emission spectra under deoxygenated condition of polymer **1** in $CHCl_3$ before irradiation (red), after 4 hours irradiation (blue), and after 8 hours irradiation (green).

Time-course of depolymerization

Depolymerization were proceeded in both the insulated and uninsulated polymers (Figure S7). However, the emission in the solid state was dramatically changed by the supramolecular strucutres. The emission behaviors were changed among orange-white-blue in the insulated polymer, while changed from dark to blue in the uninsulated polymer (Figure 3a-3g and Figure S8).

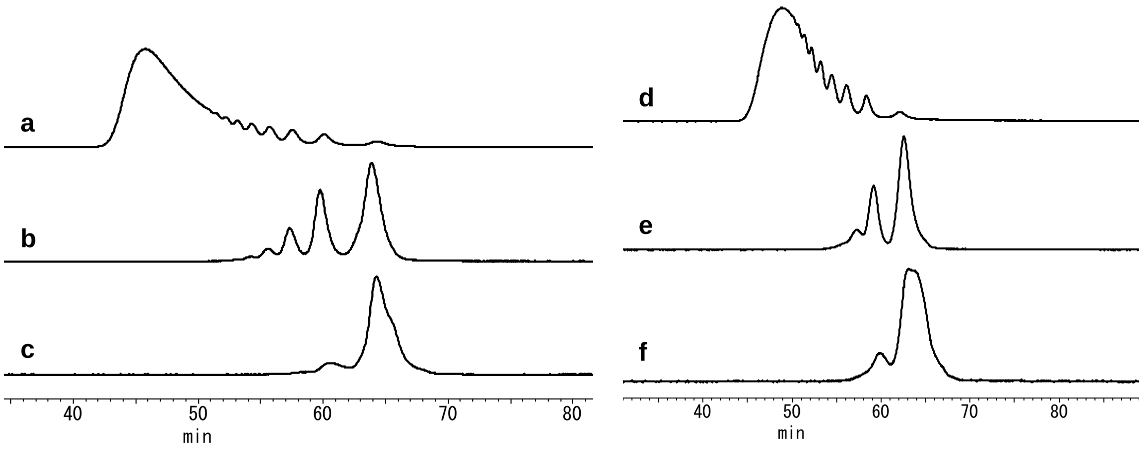

Figure S7. SEC analyses (detected: UV 380 nm) of the insulated polymer **1** with HCl gas; (a) before reaction, (b) after 1.5 h, and (c) after 4 h, and of the uninsulated polymer **2** with HCl gas; (d) before reaction, (e) after 10 min, and (f) after 30 min.

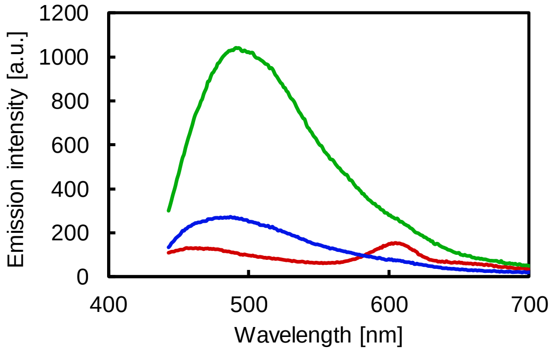

Figure S8. Emission spectra under deoxygenated condition of uninsulated polymer (**2**) films on SiO_2 substrates reacted with HCl gas before reaction (red), after 10 min (blue), and after 30 min (green).

Gas selectivity

The depolymerizing reactions of insulated polymer **1** were conducted at room temperature under the target gas (N_2, CO_2, CH_4, dry air, HCl, CO, NO, O_2, H_2, C_2H_4, NH_3, and H_2S). After 3 h, emission spectra of the resultant substrate was measured under deoxygenated condition. Figure S9 displayed the emission spectra of each resultant substrate. The ratios of the emission intensities at 460 nm and 583 nm against each target gas were shown in Figure 3h. After reaction, the polymer films were dissolved in THF and then analyzed by SEC. Figure S10 indicated that the emission changes were attributed to the depolymerization of the insualted polymer **1**.

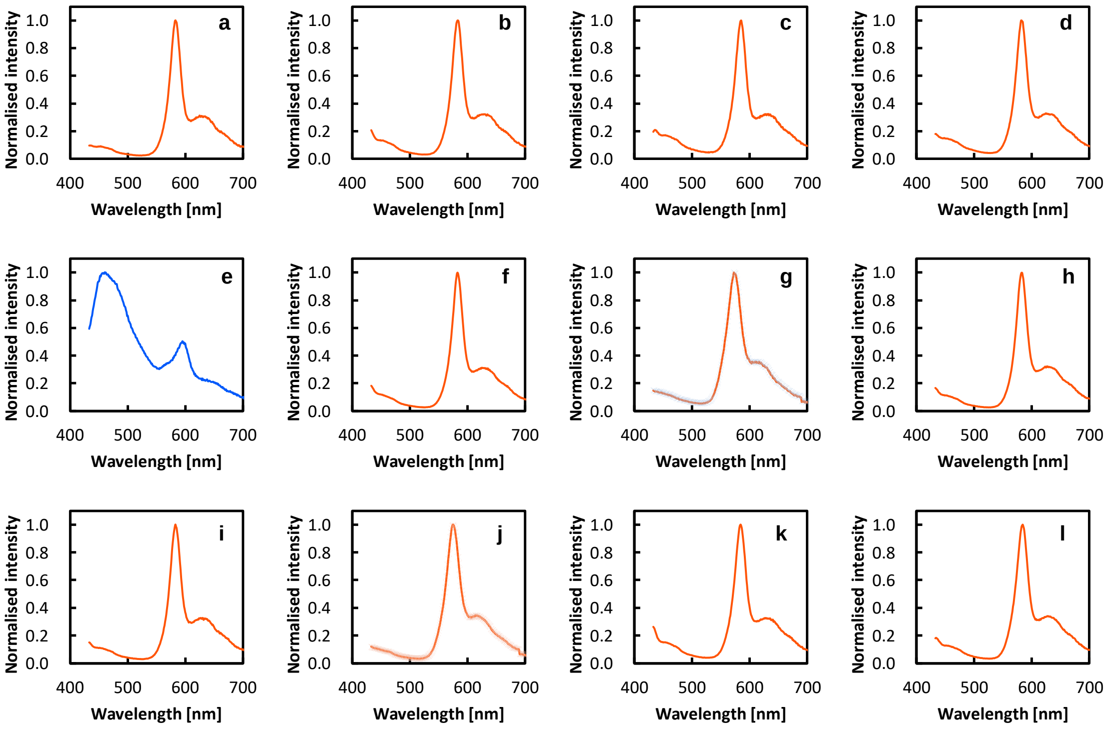

Figure S9. Emission spectra under deoxygenated condition of insulated polymer **1** on SiO_2 substrates after reactions with various gasses: (a) N_2, (b) CO_2, (c) CH_4, (d) dry air, (e) HCl, (f) CO, (g) NO, (h) O_2, (i) H_2, (j) C_2H_4, (k) NH_3, and (l) H_2S.

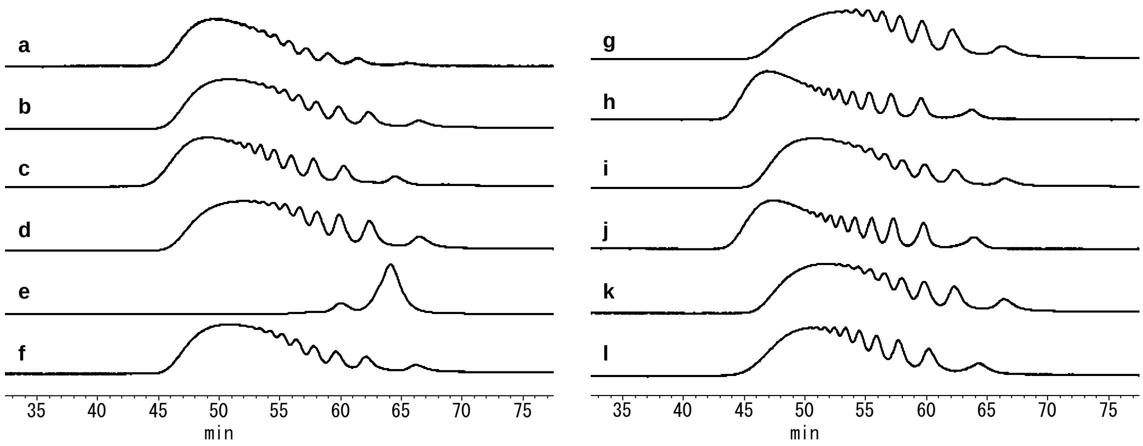

Figure S10. SEC analyses (detected: UV 380 nm) of insulated polymer **1** on SiO_2 substrates after reactions with various gasses: (a) N_2, (b) CO_2, (c) CH_4, (d) dry air, (e) HCl, (f) CO, (g) NO, (h) O_2, (i) H_2, (j) C_2H_4, (k) NH_3, and (l) H_2S.

HCl detection under ambient condition

Insulated polymer (**1**) film on SiO_2 substrate displayed orange phosphorescence even under the air condition because the cyclic insulation prohibit the quenching process with oxygen. Movie S1 demonstrated the visual change via spray of 5 mL HCl gas under air. After HCl gas exposure, the emission color dramatically changed from orange to blue (Figure S11a), indicating the color-tunability by HCl under the ambient condition. Figure S11b described the insutruments of the experiment.

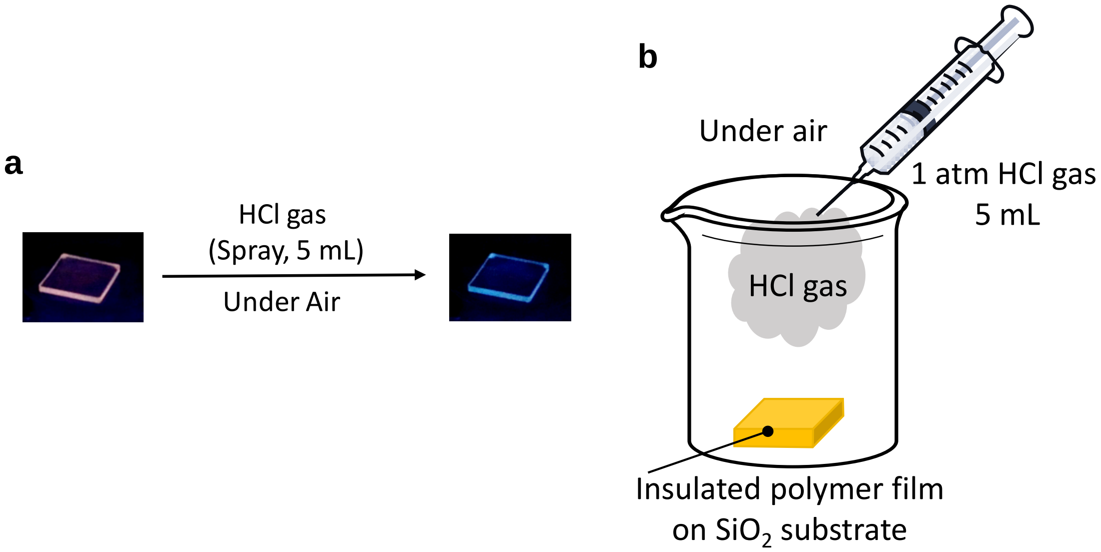

Figure S11. (a) Photographic images under 365 nm excitation (under air) of insulated polymer (**1**) film before and after the reaction with HCl gas spray. (b) Illustration of the experiment of HCl gas spray to insulated polymer (**1**) film under air.

2-3. Ligth-induced acceleration of HCl reaction

Control experiment

Insulated polymer (**1**) film on a SiO_2 substrate added into 100 mL two-necked round-bottom flask (200 mL total volume). The flask was filled with N_2 gas, and then HCl gas (10 mL or 20 mL) was added into the flask to provide the desired concentrations (5%v/v or 10%v/v, respectively). The flask was kept under UV irradiation (350–400 nm) with MAX-303 (Asahi Spectra) bearing mirror module (250–385 nm) and cutoff filters at room temeprature for 4 min (Figure S12d). As control experiments, the reactions were conducted at a room temperature (Figure S11a) and 100 °C (Figure S12b) without UV irradiation, or at a room temperature under UV irradiation (450–500 nm) with MAX-303 (Asahi Spectra) bearing mirror module (385-740 nm) and cutoff filters (Figure S12c).

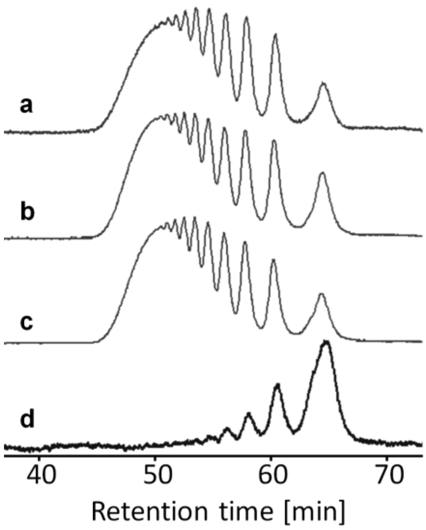

Figure S12. SEC analyses (detected: UV 380 nm) of insulated polymer **1** on SiO_2 substrates under 5%v/v HCl gas with various reaction conditions; (a) at room temperature without UV irradiation, (b) at 100 °C without UV irradiation, (c) at room temperature with 450–500 nm excitation, and (d) at room temperature with 350–400 nm excitation.

Site-selective irradiation

Insulated polymer film on a SiO_2 substrate reacted with 10%v/v HCl gas for 1 min with spot irradiations under UV irradiation (350–400 nm) with MAX-303 (Asahi Spectra) bearing mirror module (250–385 nm) and cutoff filters. Figure 4c displayed the irradiation pattern of the spots. The resultant substrate after reaction indicated the the emission color only at the irradiated area was changed from orange phosphorescence to blue fluorescence (Figure 4b). These results demenostrated that the UV light clearly accelerated the depolymerizing reaction of insulated polymer with 10%v/v HCl gas.

Experiments for low HCl concentration in the solution

Insulated polymer **1** (0.05 mg) was desolved in MeOH/H_2O (2/1) (total volume: 2.0 mL) with 12 M hydrochloric acid (23 μL) to provide the desired concentrations (5000 ppm), and then the mixture was kept under UV irradiation with SLUV-4 (ASONE) at room temperature for 1 h. The light-induced accerelation successfully visualized such low-concentrated HCl gas (Figure S13).

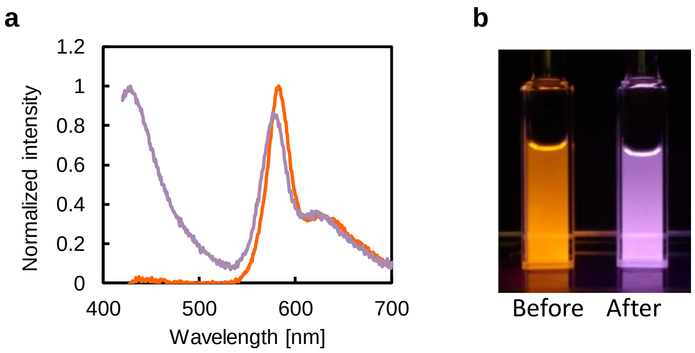

Figure S13. (a) Emission spectra and (b) photographic images of the reaction mixture in MeOH/H_2O (2/1) with insulated polymer before (orange) and after (gray) reaction with 5000 ppm HCl under UV irradiation.

Experiments for low HCl concentration in the solid state

Insulated polymer film on a SiO_2 substrate added into 100 mL two-necked round-bottom flask (200 mL total volume). The flask was filled with N_2 gas, and then HCl gas (0.1 mL) was added into the flask to provide the desired concentrations (500 ppm). The flask was kept under UV irradiation (350–400 nm) with MAX-303 (Asahi Spectra) bearing mirror module (250–385 nm) and cutoff filters at room temeprature for 2 min. As a result, the light-induced acceleration of polymer film was confirmed even under low HCl concentration and short reaction time (Figure 4d and S14).

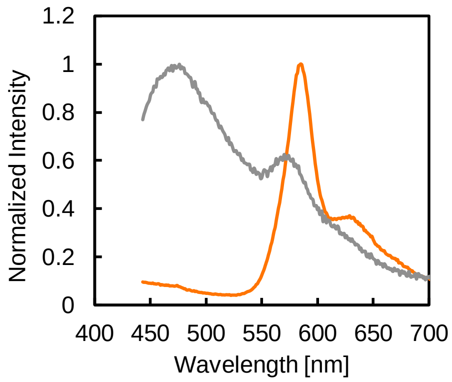

Figure S14. Emission spectra of the reaction mixture in MeOH/H_2O (2/1) with insulated polymer before (orange) and after (gray) reaction with 500 ppm HCl under UV irradiation.

^{31}P NMR analyses for complex 4

Complex **4** (5.1 mg) was desolved in DMF (30 mL) with 12 M hydrochloric acid (2.5 mL) to provide the desired concentrations (1 M), and then the mixture was bubbled with nitrogen gas for 10 min in order to remove oxygen gas form the system. UV irradiation (365 nm, 70 mW/cm^2, 20 min) was performed at a distance of 2 mm using a wide-angle LED lamp (HLV-24UV365-4WNRBT, CCS Inc.). The whole system was cooled by a fan to keep room temperature. The mixture was diluted with EtOAc and washed with brine and $NaHCO_3$ aq.. The organic layer was separated and dried over $MgSO_4$, and then filtered. The solvent was removed by evaporation. The residue was dissolved in $CDCl_3$ and analyzed by ^{31}P NMR. The results were shown in Figure 5b in the manuscript.

Additional note: Control experiments under single stimulus, either addition of HCl or UV irradiation, were conducted with the same experimental procedure. As the control experiment with UV, water (2.5 mL) was added instead of hydrochloric acid into the solution of **4** in DMF.

2-4. NMR spectra of 4

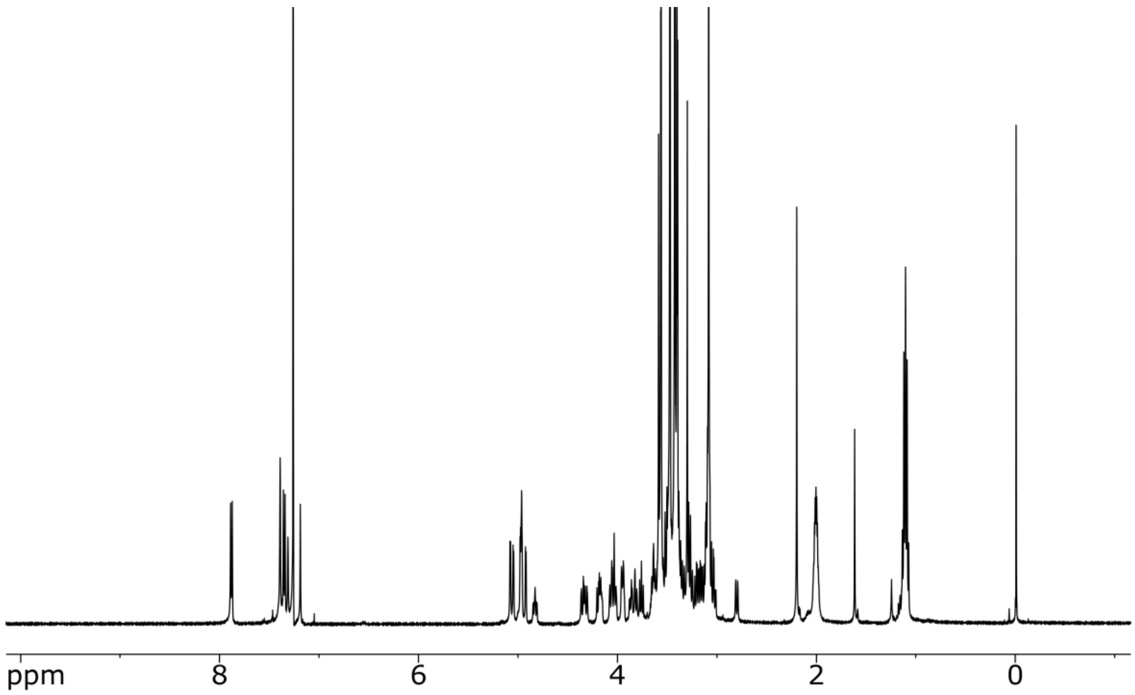

Figure S15. ^{1}H NMR spectrum of **4** (500 MHz, $CDCl_3$)

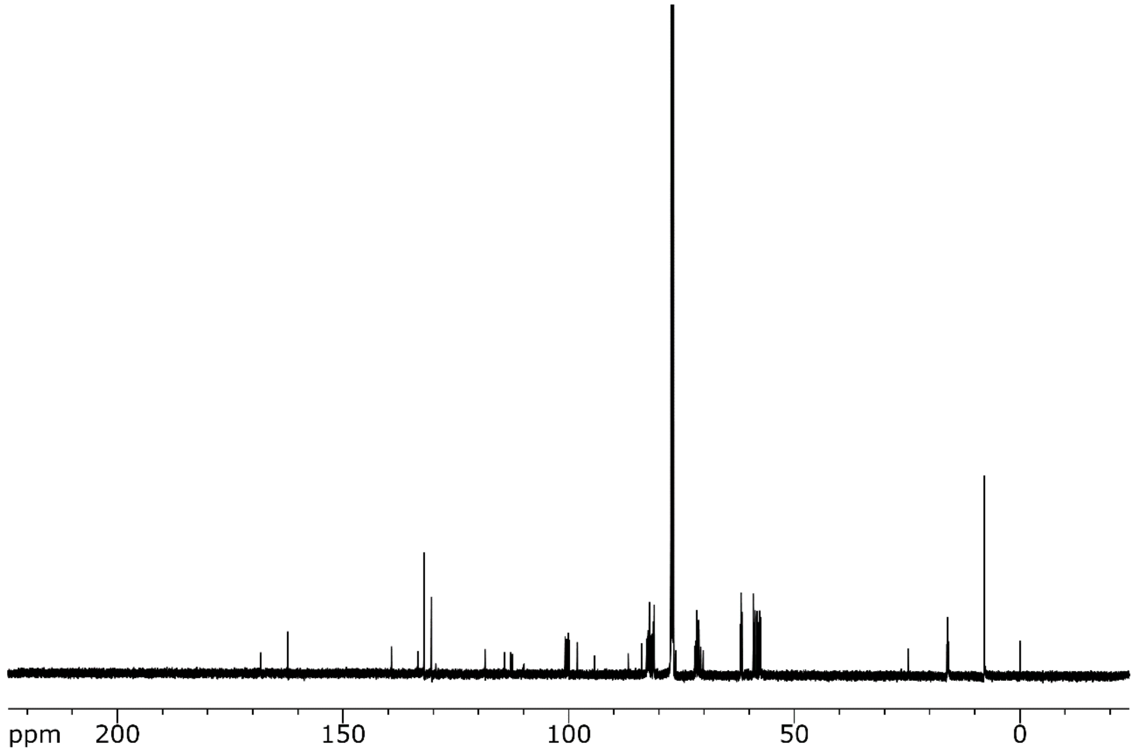

Figure S16. ^{13}C NMR spectrum of **4** (126 MHz, $CDCl_3$)

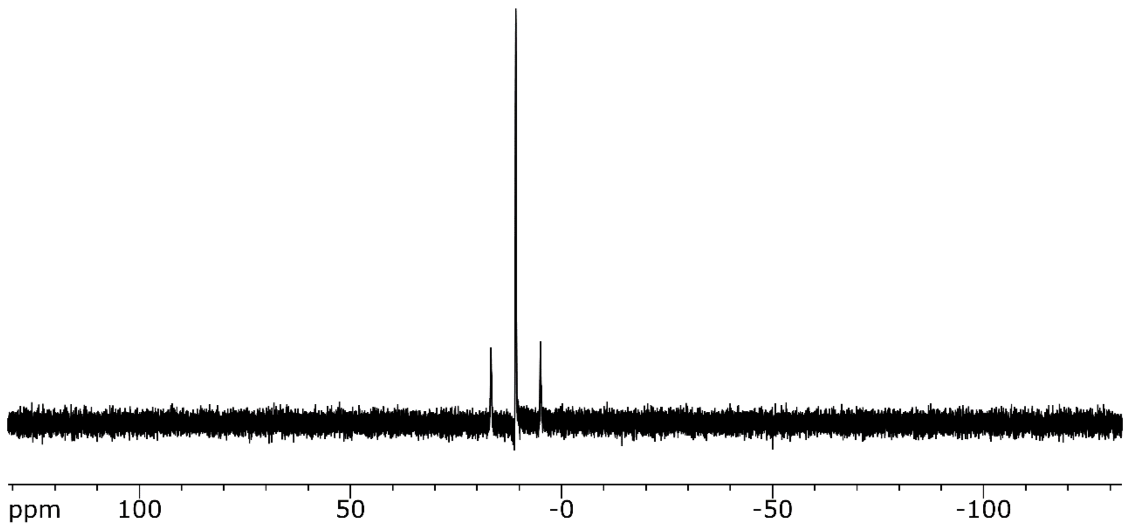

Figure S17. ^{31}P NMR spectrum of **4** (202 MHz, $CDCl_3$)

3. References

[1] Rahn, J. A.; Nelson, J. H.; Baltusis, L. Solid-state structures of $(R_3P)_2PtX_2$ complexes As determined by a combination of $^{13}C\{^1H\}$ and $^{31}P\{^1H\}$ NMR spectroscopy. *Inorg. Chem.* **1990**, 29, 750–755.

[2] Sebald, A.; Stader, C.; Wrackmeyer, B.; Bensch, W. Alkynyl/chloride exchange between trans-platinum(II) and -palladium(II) chlorides and alkynylstannanes. Crystal structure of trans-[bis(1-propynyl)-bis(triethylphosphine)platinum(II)]. *J. Organomet. Chem.* **1986**, 311, 233–242.
